# Genetic Analysis in Maize Foundation Parents with Mapping Population and Testcross Population: Ye478 Carried More Favorable Alleles and Using QTL Information Could Improve Foundation Parents

**DOI:** 10.3389/fpls.2016.01417

**Published:** 2016-09-23

**Authors:** Yinghong Liu, Xianbin Hou, Qianlin Xiao, Qiang Yi, Shaowei Bian, Yufeng Hu, Hanmei Liu, Junjie Zhang, Xiaoqin Hao, Weidong Cheng, Yu Li, Yubi Huang

**Affiliations:** ^1^Maize Research Institute, Sichuan Agricultural UniversityChengdu, China; ^2^College of Agronomy, Sichuan Agricultural UniversityChengdu, China; ^3^College of Life Science, Sichuan Agricultural UniversityYa'an, China; ^4^College of Agronomy, Guangxi UniversityNanning, China; ^5^Maize Research Institute, Guangxi Academy of Agricultural SciencesNanning, China; ^6^Institute of Crop Science, Chinese Academy of Agricultural SciencesBeijing, China

**Keywords:** maize, foundation parent, quantitative trait loci, general combining ability, improvement

## Abstract

The development of maize foundation parents is an important part of genetics and breeding research, and applying new genetic information to produce foundation parents has been challenging. In this study, we focused on quantitative trait loci (QTLs) and general combining ability (GCA) of Ye478, a widely used foundation parent in China. We developed three sets of populations for QTL mapping and to analyze the GCA for some agronomic traits. The assessment of 15 traits resulted in the detection of 251 QTLs in six tested environments, with 119 QTLs identified through a joint analysis across all environments. Further, analyses revealed that most favorable alleles for plant type-related traits were from Ye478, and more than half of the favorable alleles for yield-related traits were from R08, another foundation parent used in southwestern China, suggesting that different types of foundation parents carried different favorable alleles. We observed that the GCA for most traits (e.g., plant height and 100-kernel weight) was maintained in the inbred lines descended from the foundation parents. Additionally, the continuous improvement in the GCA of the descendants of the foundation parents was consistent with the main trend in maize breeding programs. We identified three significant genomic regions that were highly conserved in three Ye478 descendants, including the stable QTL for plant height. The GCA for the traits in the F_7_ generation revealed that the QTLs for the given traits *per se* were affected by additive effects in the same way in different populations.

## Introduction

Maize (*Zea mays* L.) is the most widely grown crop in China, with the average yield increasing from 2.09 tons/ha in 1970 to 5.81 tons/ha in 2014 (http://www.stats.gov.cn/). Several studies proposed that more than 50% of this increase in China was due to genetic improvement (Zhang et al., 1998; Xie et al., 2009; Ci et al., 2011; Niu et al., 2013), which was similar to the findings of studies in other countries (reviewed in Duvick, 2005). Because single-cross varieties cover almost all total planting areas of maize in China, foundation parents, which exhibit excellent agronomic characteristics, and high general combining ability for yield-related traits, are crucial for maize breeding (Troyer, 1999, 2004; Duvick et al., 2004; Teng et al., 2004; Hallauer and Carena, 2009; Li, 2009; Li and Wang, 2010). Li and Wang (2010) proposed a quantitative definition of foundation parents that they have at least 20 descended inbred lines and 30 important hybrids which used the given foundation parent as one of the two direct parent. Many studies have provided useful information regarding the generation of foundation parents in different germplasm background. Analyses of their pedigrees revealed that foundation parents at early stage were derived from a few conventional cultivars or landraces, while those foundation parents developed later were from crosses between elite lines or from recurrent selection populations (Smith et al., 1990; Troyer, 1999, 2004; Mickel and Dudley, 2006; Mikel, 2006, 2011; Li and Wang, 2010). For morphology traits and physiology traits studies of foundation parents contributed to divide the heterotic group and guide breeding (because hybrids that its parents from two heterotic groups have greater hybrid vigor than hybrids whose parents are both from one heterotic group) (Smith and Smith, 1989; Duvick et al., 2004; D'Andrea et al., 2006; Li, 2009; Ci, 2011; Liu Y. H. et al., 2011). Studies examining combining abilities (the ability to combine with testers) produced abundant genetic information that divided the heterotic group, and allowed for the evaluation of the breeding value of inbred lines (Beck et al., 1991; Li et al., 2002; Makumbi et al., 2011).

Molecular markers have been used to study the genetic diversity of maize inbred lines, resulting in useful information regarding maize population structures and genomic distributions of linkage disequilibrium (Ching et al., 2002; Liu et al., 2003; Reif et al., 2005; Stich et al., 2005; Wang et al., 2008; Van Inghelandt et al., 2010; Wu et al., 2014). These studies have revealed that there are conserved genetic regions in foundation parents and their descendants. Investigations of maize genetic architecture of important traits using quantitative trait locus (QTL) mapping indicated that foundation parents consist of several favorable alleles or allele combinations (Frascaroli et al., 2007; Buckler et al., 2009; Liu W. X. et al., 2011). Additionally, research regarding the development of foundation parents involving genomics, transcriptomics, proteomics, and metabolomics has determined the importance of the enrichment of rare alleles and improved genetic balance (Duvick et al., 2004; Chen Z. Y. et al., 2007; Li et al., 2009; Jiao et al., 2012; Rao et al., 2014).

Foundation parents represent a comprehensive concept from which breeding practices were derived. These parents were not only used to make hybrids with other inbred lines, they were also applied for the generation of diverse descendants for subsequent breeding cycles. Therefore, studying the appearance of maize foundation parents must be associated with its utility for breeding programs. Many of the studies mentioned above attempted to explain how maize foundation parents formed, but did not focus on the relevance to maize breeding in the future. Quantitative trait locus mapping of complex traits may provide abundant information regarding genetic architecture and molecular markers of favorable alleles for molecular breeding (Yin et al., 2003; Cai, 2006; Buckler et al., 2009; Xu, 2010; Liu Y. et al., 2014). And many statistical methods have been proposed and developed to map multiple traits and providing more details on the genetic architecture of complex traits (Malosetti et al., 2008; Silva et al., 2012; Scutari et al., 2014). A test of general combining ability (GCA, the average ability to combine with testers) will also help determine the breeding value of inbred lines (reviewed in Duvick, 2005; Hallauer et al., 2010a). Integrating QTL mapping with a test of combining ability may lead to a better understanding of the development and improvement of maize foundation parents.

The objectives of this study were to (1) identify the favorable alleles that had been pyramided in the foundation parent “Ye478,” a famous parent in China, using 266 R08 × Ye478 F_2:3_ families, (2) assess the improvement of foundation parents using Ye478-related lines (Ye478's parents and its descendants), and (3) determine the utility of QTL information for traits *per se* for improving of foundation parents by analyzing 36 R08 × Ye478 F_7_ generations. Our results will provide valuable information for developing and improving elite maize lines in breeding programs.

## Materials and methods

### Plant materials

The mapping population (MP) consisted of 266 F_2:3_ families derived from a cross between R08 and Ye478, which were the foundation parents widely the southwest maize zone and the Huanghuaihai maize zone of China (mainly summer maize region), respectively. More than 20 important hybrids with R08 as a parent have been released (Qiao et al., 2009). More than 30 inbred lines and 58 important hybrids with Ye478 as a parent have been released (Li and Wang, 2010).

A testcross population (TCP1) for examining the improvement of Ye478 was developed using six Ye478-related inbred lines crossed with five testers according to the North Carolina Design II mating design (Comstock and Robinson, 1948). The Ye478-related inbred lines included two parental lines, i.e., Shen5003 and U8112 and three descended inbred lines, namely Zheng58, Zao48, and K22 (Supplemental Table 1). The five testers were Chang7-2, Mo17, S37, Qi319, and Dan 340, which belong to different heterotic groups (i.e., Tang Si Ping Tou, Lancaster, Suwan, PB, and Luda Red Cob) (Xie et al., 2007).

Another testcross population (TCP2), including 190 testcrosses, for analyzing the effects of molecular-assisted selection on foundation parents was obtained by crossing R08, Ye478, and 36 F_7_ lines of R08 × Ye478 with the above-mentioned five testers according to the North Carolina Design II mating design.

### Field experiments

The F_2:3_ families of R08 × Ye478 along with the parental lines and the F_1_ hybrid were evaluated in 2012 and 2013 at the following locations in China: The Duoying farm at the Maize Research Institute of Sichuan Agricultural University, Ya'an (EY; 30°N, 103°E), the Xishuangbanna maize breeding site at the Maize Research Institute of Sichuan Agricultural University, Jinghong (EJ; 22°N, 100.5°E), and the Maize Research Institute of Guangxi, Nanning (EN; 22.5°N, 108.2°E), Guangxi Zhuang Autonomous Region. Field experiments were completed according to a randomized complete block design, with one-row plots and two replicates at each site. Rows were 3 m long, with a 0.8-m space between rows, for a final density of 58,000 plants/ha. Fields were managed according to local practices.

The TCP1 plants were evaluated in 2013 and 2014 at the Duoying farm and Xishuangbanna maize breeding site. The experiments were conducted using a randomized complete block design involving one-row plots and three replicates at each location. The TCP2 plants were evaluated in 2014 at the same two locations, using a similar experimental design, but with two replicates per site. Cultivation practices were the same as those used for the MP plants.

Each combination of location and year was considered as an individual experimental environment, and each environment was named using an abbreviation derived from the location and year [i.e., EY13 refers to Ya'an (environment) in 2013].

### Phenotypic analyses

Five competitive plants from the middle of each plot were used in evaluation of the following nine plant type-related traits: Plant height (PH, cm), ear height (EH, cm), internode length above the primary ear (IL, cm), tassel length (TL, cm), leaf number above the ear (LN), tassel branch number (TB), leaf length above/below the primary ear (LLA/B, cm), leaf width above/below the primary ear (LWA/B, cm), leaf angle above/below the primary ear (LAA/B, degree), length from leaf collar to flagging point above/below the primary ear (Lf, cm), and leaf orientation value above and below the primary ear (LOV). Additional five competitive plants were harvested from the middle of each plot to assess the following yield traits: Ear length (EL, cm), ear diameter (ED, cm), ear-cob diameter (CD, cm), ear row number (ER), and 100-kernel weight (KW, g). The trait measurement methods are described in Supplemental Table 2. All traits were evaluated in the F_2:3_ family plants, while all traits except for LN were analyzed in the TCP1 and TCP2 plants.

Analysis of variance was completed with PROC GLM using SPSS (http://www.spss.com). Broad-sense heritability ($H_{B}^{\text{2}}$) and its confidence intervals were calculated as described by Hallauer et al. (2010c) using the following equations: $H_{B}^{\text{2}}$ = $\sigma_{\text{g}}^{2}$/($\sigma_{\text{g}}^{2}\;+$ $\sigma_{\text{ge}}^{2}$/n + σ_2_/nb) (MP plants) and $H_{B}^{\text{2}}$ = $\sigma_{\text{m}}^{2}$/($\sigma_{\text{m}}^{2}$ + $\sigma_{\text{fm}}^{2}$/f + $\sigma_{\text{em}}^{2}$/n + $\sigma_{\text{efm}}^{2}$/nf + σ_2_/nbf) (TCP plants), where $\sigma_{\text{g}}^{2}$ is the genetic variance, $\sigma_{\text{ge}}^{2}$ is the genotype × environment interaction variance, σ^2^ is the error variance, n is the number of environments, b is the number of replicates per experiment; $\sigma_{\text{m}}^{2}$ is the male variance, $\sigma_{\text{fm}}^{2}$ is the female × male interaction variance, $\sigma_{\text{em}}^{2}$ is the environment × male interaction variance, $\sigma_{\text{efm}}^{2}$ is the environment × female × male interaction variance, and f is the number of females. The $H_{B}^{\text{2}}$ confidence intervals were estimated according to the method proposed by Knapp et al. (1985). Additive (σ) variances were estimated using the procedure established by Hallauer et al. (2010b). The Pearson's phenotypic or GCA correlations were determined using SPSS PROC CORR (http://www.spss.com).

### Molecular linkage construction and QTL mapping

Genomic DNA was extracted from young leaves of the F_2:3_ plants and their parents (at least 10 plants per line as a bulk sample) and F_7_ lines (five plants per line as a bulk sample) using the modified procedure involving cetyltrimethylammonium bromide (Chen and Ronald, 1999). The oligonucleotide pool assay used in this study was developed by the National Maize Improvement Center of China using Illumina GoldenGate technology. This assay consisted of 3072 well-distributed and high-quality (i.e., high calling rate, polymorphism rate, and minor allele frequency) single nucleotide polymorphisms (SNPs) selected from 56,110 SNPs in 513 maize inbred lines. Genotyping was completed using an Illumina BeadStation 500 G (Illumina, San Diego, CA, USA) at the National Maize Improvement Center of China according to a published protocol (Fan et al., 2006). Chi-squared analyses of the segregation ratio of each SNP in the F_2:3_ families were conducted at a significance threshold of 5%, and then molecular markers affected by segregation distortion were excluded. Ultimately, 471 polymorphic markers were used to construct a genetic linkage map, which agreed with the expected Mendelian segregation ratio of 1:2:1. The genetic map was developed using MAPMAKER/EXP version 3.0b, with a logarithm of odds threshold > 3.0 (Lander et al., 1987). The Kosambi mapping function was used to convert recombination frequencies to genetic distances (Kosambi, 1943).

The QTL locations, origins of positive alleles, and QTL effects on each trait for each environment (i.e., single environment analysis, SEA) were investigated, and a joint analysis across all environments (JAAE) was completed, using the QTL Network software version 2.1 (Yang J. et al., 2008) with a mixed model-based composite interval mapping method (Wang et al., 1999; Yang et al., 2007). The genome scan configuration used a 10-cM testing window and a 1-cM walk speed to identify QTLs associated with traits, and a 10-cM filtration window to distinguish between two adjacent test statistic peaks (whether they are two QTL or not). The threshold for identifying a significant QTL was defined by 1000 permutations (*P* < 0.05) (Churchill and Doerge, 1994). The QTLs detected in different environments for the same trait were considered to be the same if their confidence intervals overlapped.

The additive effects of favorable alleles can improve plant morphology to enable adaptation to high plant densities or enhance yield (Mock and Pearce, 1975; Duvick, 2005; Tollenaar and Lee, 2011). Therefore, alleles were considered favorable if they had a positive additive effect on IL, LOV, EL, ED, ER, and KW, or if they had a negative additive effect on PH, EH, TL, LN, TB, LL (leaf length), LW (leaf width), LA (leaf angle), and CD. The contribution of Ye478 to favorable effects was calculated using the following formula: FOY = $\overset{\text{j}}{\underset{\text{n}}{\sum }}|\text{aj}|/\overset{\text{i}}{\underset{\text{N}}{\sum }}|\text{ai}|$, where aj is the additive effect of QTLs with a favorable Ye478 allele, and ai is the additive effect of all QTLs.

### Calculation of general combining ability

The additive-dominance genetic model (Zhu, 1992; Zhu and Weir, 1994) was used to calculate the GCA for all traits using the QGA Station software (http://mypage.zju.edu.cn/Jun_Zhu). The y phenotype value was defined as y = μ + A + D + E + AE + DE + e, where μ is the population mean, A is the additive effect (GCA), D is the dominance effect (key component of specific combining ability), E is the environment effect, AE is the additive × environment interaction effect, DE is the dominance × environment interaction effect, and e is the residual effect. Genetic correlation coefficients were estimated using the minimum norm quadratic unbiased estimation method. Jackknifing (i.e., cutting one genotype once) was used to approximate the standard errors of the estimated genetic parameters (Miller, 1974; Zhu and Weir, 1994). A *t* test (two-tailed) was used to test the significance of the genetic parameters.

## Results

### Quantitative trait loci detected using the mapping population and pyramiding of favorable alleles in Ye478/R08

Results for the $H_{B}^{\text{2}}$, linkage map, and QTL analysis for the 15 traits in the F_2:3_ families are presented in Table 1, Supplemental Figure 1, Supplemental Tables 3, 4. We detected 251 putative QTLs for the 15 traits in six environments (Supplemental Figure 2). There were five (ED) to 30 (TB) QTLs for single traits in the six environments. Additionally, in EY13 and EY12, a total of 29 and 39 QTLs, respectively, were identified for all the 15 traits. For an individual trait in a single environment, there were 0–6 QTLs. Over half of the alleles had favorable additive effects from Ye478 for the plant type-related traits, except for IL, TB, and LAA. Similar results were observed for the contribution of Ye478 to the favorable effect (the FOY ratio). In all environments, over half the favorable alleles were contributed by Ye478, and in most environments, the FOY values exceeded 50%. Moreover, the FOY values for ER and KW were over 50%.

**Table 1:** **Quantitative trait loci detected in the F_2:3_ populations**.

	**Single environment analysis**						**Stable QTL**	**Joint analysis across all environment**	**Important QTL**
	**EN12**	**EN13**	**EY12**	**EY13**	**EJ12**	**EJ13**			
PH	3/0/100	2/1/80.1	5/0/100	3/0/100	3/0/100	4/0/100	4/0/-	7/3/82.8	6/0/100
EH	4/0/100	1/0/100	2/0/100	1/1/57.0	2/1/78.1	4/0/100	4/0/-	5/0/100	4/0/100
IL	1/2/27.8	0/1/0	0/1/0	0/1/0	0/1/0	0/1/0	0/2/-	4/3/38.0	0/3/0
TL	3/0/100	2/0/100	1/0/100	1/0/100	4/0/100	4/0/100	3/0/-	5/0/100	3/0/100
LN	1/2/42.0	1/1/51.1	3/0/100	1/0/100	2/1/69.8	0/1/0	3/2/-	3/2/74.1	3/1/80.5
TB	3/2/44.1	0/4/0	1/4/27.5	2/3/45.4	1/4/19.7	2/4/30.0	2/5/-	3/6/28.7	1/5/17.5
LLA	2/1/64.9	3/1/76.8	2/0/100	2/0/100	1/1/60.1	1/0/100	2/1/-	3/1/86.7	2/0/100
LWA	0/3/0	1/3/24.6	3/2/64.1	3/0/100	3/0/100	2/1/62.2	4/2/-	5/4/58.7	3/4/49.3
LAA	0/0/-	1/1/40.0	2/2/59.7	0/1/0	0/0/-	1/2/36.9	1/1/-	2/2/54.0	3/1/72.4
LOV	0/0/-	2/0/100	2/0/100	2/0/100	1/0/100	2/0/100	3/0/-	4/4/57.4	2/0/100
EL	1/2/15.8	1/1/38.8	2/1/47.6	0/1/0	0/2/0	0/1/0	0/1/-	0/1/0	0/1/0
ED	0/0/-	1/0/100	0/1/0	1/0/100	1/1/45.2	0/0/-	0/0/-	2/2/48.2	2/1/64.7
CD	1/0/100	1/2/44.7	0/0/-	1/0/100	0/1/0	0/0/-	1/0/-	1/2/43.2	1/1/54.5
ER	1/0/100	2/1/68.2	2/3/37.6	3/1/61.4	1/2/37.0	1/1/43.8	2/2/-	4/3/58.7	3/2/63.6
KW	1/1/50.8	1/1/59.3	0/0/-	1/0/100	1/0/100	0/1/0	0/0/-	4/2/79.1	3/1/84.2

*The abbreviations EN, EY, and EJ refer to Nanning, Ya'an, and Jinghong, respectively. The 12 and 13 refer to 2012 and 2013, respectively. The abbreviations PH, EH, IL, TL, LN, TB, LLA, LWA, LAA, LOV, EL, ED, CD, ER, and KW refer to plant height, ear height, internode length above the primary ear, tassel length, leaf number above the ear, tassel branch number, leaf length above the primary ear, leaf width above the primary ear, leaf angle above the primary ear, leaf orientation value, ear length, ear diameter, ear-cob diameter, ear row number, and 100-kernel weight, respectively. The values presented as “#/#/#” correspond to the number of favorable alleles from Ye478, the number of favorable alleles from R08, and the contribution of Ye478 to the favorable effect, respectively. The contribution of Ye478 to the favorable effect was calculated using the formula FOY = $=\sum_{n}^{j}|aj|/\sum_{N}^{i}|ai|$, where aj is the additive effect of QTLs with a favorable Ye478 allele, and ai is the additive effect of all QTLs*.

A total of 45 putative stable QTLs across environments (SQ; i.e., QTL was detected in at least two environments) were identified for all the 15 traits (Table 1, the stable QTL column). The number of single trait SQs ranged from zero (ED and KW) to seven (TB). We detected 56 putative important QTLs [i.e., QTL was detected by JAAE (*R*^2^ > 2%) in at least one environment] for all 15 traits (Table 1, the last column). No fewer than 50% of SQ alleles that had favorable additive effects were from Ye478 (except for IL, TB, and EL). A similar result was observed for the important QTLs.

### Favorable dwarfism alleles from Ye478

Semi-dwarfism is an important agronomic trait that enables the wide use of Ye478 in China (Li and Wang, 2010). We analyzed the pyramiding of favorable alleles in Ye478, and detected 13 SQs and 12 major QTLs (MQ; i.e., QTLs with *R*^2^ > 10% in at least one environment, and also identified based on joint analysis across six environments) associated with four plant height-related traits (Table 2, Supplemental Table 4, 4 SQs related to PH, 4 SQs related to EH, 2 SQs related to IL and 3 SQs related to TL).

**Table 2:** **Quantitative trait loci mapping results *via* joint analysis across all environments for plant height-related traits in the F_2:3_ populations**.

**QTL**	**Env**.	**Interval**	**Site(cM)**	**Range(cM)**	**A**	**D**	**R^2^(%)**
**PH**							
*QJPH1*	5	SYN275/PZE-101213558	267.7	264.1–270.7	−5.64		11.6
*QJPH2*	5	PZE-102146058/PZE-102178263	180.3	179.8–181.3	−6.31		6.6
*QJPH5-1*	3	PZE-105123635/SYN20663	88.3	85.5–94.8	−4.18		16.3
*QJPH5-2*	1	PZE-105136417/PZE-105142633	118	112.0–118.1	−2.52	2.97	14.5
*QJPH7*	1	PZE-107055832/PZE-107060597	97.9	96.0–100.6	−5.61	4.06	11.6
*QJPH8*	2	SYN22840/PZE-108016169	35.1	33.2-37.1	−5.06		8.1
Total							68.7
**EH**							
*QJEH1-1*	1	PZE-101071273/SYN6888	124	121.0–124.9	−0.59		2.1
*QJEH1-2*	4	PZE-101196709/SYN275	263.1	260.1–269.7	−3.15	−0.79	9.7
*QJEH5*	4	PZE-105132845/PZE-105132778	104.5	99.5–111.1	−3.61	1.57	15.1
*QJEH7*	1	PZE-107020363/SYN38007	87.3	82.6-89.3	−3.07		10.3
*QJEH8*	2	SYN22840/PZE-108016169	35.1	31.2–38.1	−2.67		7.4
Total							44.6
**IL**							
*QJIL5*	1	PZE-105128589/PZE-105132845	102.4	100.4–103.5	−0.46	0.21	9.9
*QJIL7*	1	PZE-107075781/PZE-107081254	117.6	114.6–123.0	−0.34	0.33	5.3
*QJIL8*	2	PZE-108068136/PZE-108110152	107.2	99.8–112.2	−0.56	−0.19	7.6
Total							22.8
**TL**							
*QJTL1*	6	PZE-101213558/PZE-101219724	276.1	273.1–278.8	−1.22		11.4
*QJTL2*	2	PZE-102178263/PZE-102149656	182.3	181.3–183.3	−1.34		9.4
*QJTL7*	1	PZB01617.2/SYN24186	77.4	67.4–83.6	−0.72	0.52	5
Total							25.8

*The abbreviations PH, EH, IL, and TL refer to plant height, ear height, internode length above the primary ear, and tassel length, respectively. Only QTLs that were consistently detected in one or more environment during single environment analyses, with R^2^ > 2% based on joint analysis across six environments are reported. The QTL names consist of three parts. The first part (QJ) refers to a QTL based on joint analysis across six environments. The second part contains an abbreviation for a trait. The number that forms the third part refers to the chromosome and serial numbers for the QTL (Supplemental Table 4)*.

Four SQs and three MQs for PH were detected on chromosomes 1, 2, 5, and 8, and four alleles that decreased PH were all from Ye478. The QTL *QSPH5* on chromosome 5 was a plant height MQ detected across four environments, and was responsible for 12.9 − 22.3% of the phenotypic variation. The QTL *QSPH2* on chromosome 2 was the only SQ detected across five environments, and was responsible for 5.8–7.4% of the observed phenotypic variation. Four SQs and four MQs for EH were detected on chromosomes 1, 5, 7, and 8, and four alleles that decreased EH were all from Ye478. The QTL *QSEH5* on chromosome 5 was an ear height MQ detected across four environments, and explained 14.4–17.9% of the phenotypic variation. Two SQs and three MQs for IL were detected on chromosomes 5, 7, and 8, and three alleles that reduced IL were all from Ye478. The QTL *QSIL5* on chromosome 5 was an MQ for internode length above the primary ear detected across three environments, and explained 11.0–15.4% of the phenotypic variation. The QTL *QSIL7* on chromosome 7 was an MQ which was detected in only one environment, and was responsible for 11.2% of the phenotypic variation. Three SQs and two MQs for TL were detected on chromosomes 1 and 2, and three alleles that decreased TL were all from Ye478. The QTL *QSTL1-2* on chromosome 1 was an MQ for tassel length detected across all six environments, and explained 8.1–16.3% of the observed phenotypic variation.

### Traits *per se* and GCA for traits in the Ye478-related lines

The analysis of variance for 14 traits in TCP1 revealed highly significant variations (*P* < 0.01) among environment, mean square (MS) of testers, and MS of the Ye478-related lines (Y) (Supplemental Table 5). There were no significant variations in the Y × E (environment) variance components (*P* < 0.05) for LLA, EL, ED, and CD. This result indicated that $\sigma_{\text{AY}}^{2}$ (i.e., additive variance estimated in the Ye478-related lines) was considerably lower than $\sigma_{\text{AT}}^{2}$ (i.e., additive variance estimated in test lines) for PH, EL, and KW (Supplemental Table 5, $\sigma_{\text{AY}}^{2}$/$\sigma_{\text{AT}}^{2}$ for PH, EL, and KW were 0.289, 0.085, and 0.220, respectively). This suggested that the GCA for the traits were conserved in the Ye478-related lines. In contrast, there were no obvious differences between $\sigma_{\text{AY}}^{2}$ and $\sigma_{\text{AT}}^{2}$ for TB, LWA, LAA, and ED (Supplemental Table 5, $\sigma_{\text{AY}}^{2}$/$\sigma_{\text{AT}}^{2}$ for TB, LWA, LAA, and ED were 0.772, 0.682, 1.600, and 1.250, respectively), with the possible reason that the effects on the GCA resulted from genetic improvements or unintentional selections in the Ye478-related lines by breeders.

**Figure 1:**
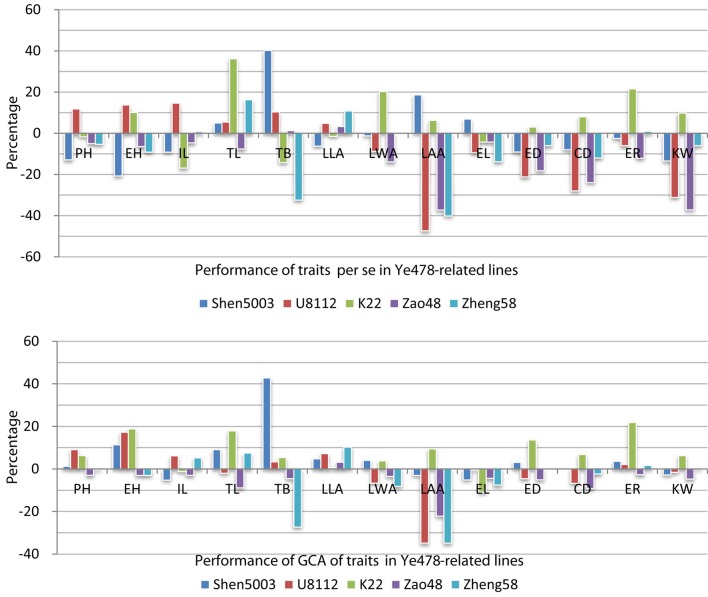
**Performance of traits per se and general combining ability for traits in the Ye478-related lines**. The abbreviations PH, EH, IL, TL, TB, LLA, LWA, LAA, EL, ED, CD, ER, and KW refer to plant height, ear height, internode length above the primary ear, tassel length, tassel branch number, leaf length above the primary ear, leaf width above the primary ear, leaf angle above the primary ear, ear length, ear diameter, ear-cob diameter, ear row number, and 100-kernel weight, respectively. The performances of traits *per se* for the Ye478-related lines are presented as relative values that were calculated with the following equation: Y_i_ = 100% × (P_i_-P_478_)/P_478_, where P_i_ refers to the phenotypic value of line i and P_478_ refers to the phenotypic value of line Ye478. The general combining ability (GCA) for traits in the Ye478-related lines are provided as relative values that were calculated with the following equation: Y_i_ = 100% × (GCA_i_- GCA_478_)/P_478_, where GCA_i_ refers to the GCA value of line i, GCA_478_ refers to the GCA value of line Ye478, and P_478_ refers to the phenotypic value of line Ye478.

Trait performance *per se* and the GCA results for the Ye478-related lines are presented in Figure 1, Supplemental Table 6. To analyze trait changes *per se* and the GCA, the values of traits *per se* and the GCA for traits were adjusted by subtracting the corresponding Ye478 value. There was considerable variance in traits *per se* for TL, TB, LAA, and KW, because all of them had more than one-line change than 30% of the Ye478. Additionally, the variability in the GCA for TB and LAA both had more than one-line change than 30% of the Ye478 (Figure 1, the first bar of TB, and the second and fifth bar of LAA). Furthermore, PH in Ye478's descendants all change no more than 10% of the Ye478, suggesting that PH was an important factor in the improvement of Ye478 (Figure 1, the third to fifth bar of PH). There were differences of five traits *per se* between Zao48 and Ye478, and the GCA data of the five traits had difference of more than 10% between K22 and Ye478. More than 75% (10/13) of the traits *per se* in Shen5003 and U8112 were significantly different from Ye478. For Ye478 and its parents, the values for some plant type-related traits *per se* in Ye478 (i.e., PH, EH, IL, LLA, and LAA) were between those of the corresponding traits in Shen5003 and U8112. However, the values for yield-related traits *per se* (except for EL) were higher in Ye478 than in Shen5003 or U8112. In the three inbred lines descended from Ye478, the values for LAA and CD for Zao48 and Zheng58 were lower than those of Ye478. Values for four yield-related traits *per se* (i.e., ED, CD, ER, and KW) were higher in K22 than in Ye478. The improvement in the GCA for the traits in the Ye478-related lines differed from that of traits *per se* in the Ye478-related lines. In particular, the GCA values for PH, EH, LLA, and ER were lower in Ye478 than in Shen5003 or U8112.

The specific combining ability (SGA) values of traits for the Ye478-related lines and testers are presented in Supplemental Table 7. The SGA effects for IL and KW were not significant (*P* < 0.05) in all F_1_ crosses, indicating that these two traits may be primarily influenced by additive effects. For Ye478 and its parents, the significant SGA effects between Ye478 and the testers were more similar to those between U8112 and the testers, than between Shen5003 and the testers. For the three inbred lines descended from Ye478, the significant SGA effect between Zheng58 and the testers was similar to that between Ye478 and the testers, while the significant SGA effects between K22 and the testers or Zao48 and the testers were not.

### Genomic regions transmitted from Ye478 to its descendants

We analyzed the transmission of significant genomic regions (SGRs; i.e., contained stable QTLs across environments for key agronomic traits) in the Ye478-related lines using genotyping data from 3072 SNPs and RNA sequencing results (http://www.maizego.org/Resources.html). We focused on genomic regions that included stable QTLs identified in the MP for PH and LA. Because PH was the typical trait of Ye478 and there were no significant differences in PH between Ye478 and its descendants, while LA was a significant breeding target in modern maize breeding and there were significant differences in LA between Ye478 and all the Ye478-related lines except K22 (Duvick, 2005; Li and Wang, 2010; Supplemental Table 6). The SGR distributions in the Ye478-related lines are shown in Table 3. For Ye478, its favorable alleles associated with PH were mainly transmitted from Shen5003, while its LA-related favorable alleles were inherited from U8112. These results indicated that Ye478 inherited advantageous traits from both parents. Zheng58 received three SGRs associated with PH from Ye478, but only one SGR related to LA. K22 and Zao48 inherited six SGRs from Ye478. We also identified three SGRs (i.e., SGR1, SGR2, and SGR3) that were present in all three Ye478 descendants, likely indicating their importance to Ye478.

**Table 3:** **Distribution of the significant genomic regions in the Ye478-related lines**.

	**SGR no**.	**Chr**.	**Physical position (bp)**	**Shen5003**	**U8112**	**Zheng58**	**K22**	**Zao48**	**Effect**
PH	SGR1	1	260149117-263740719	+^a^	−	+	+	+	N^b^
	SGR2	2	190159942-193184829	−	+	+	+	+	N
	SGR3	5	180410228-181889176	+	−	+	+	+	N
	SGR4	8	8123084-15981330	+	−	−	−	−	N
LA	SGR5	1	27502892-31976599	−	+	+	−	+	N
	SGR6	2	1780080-7954053	+	−	−	+	−	P
	SGR7	3	4712826-5439550	−	+	−	+	+	N
	SGR8	5	203520565-210387229	+	−	−	+	+	N

*The abbreviations PH and LA refer to plant height and leaf angle, respectively*.

a *+ and − indicate that the genomic region was the same or different from that of Ye478, respectively*.

b *N and P indicate that whether the Ye478 alleles in the genomic region exhibited negative or positive additive effects in the mapping population, respectively*.

### Effects of SQs on traits *per se* and the GCA for traits

The effects of SQs on traits *per se* and the GCA were calculated using a *t* test, and the results are summarized in Table 4. To increase the reliability of the results, only SQs that were detected in at least four environments were analyzed (Supplemental Table 4). Of the 14 analyzed SQs, eight (associated with PH, EH, TL, TB, LLA, and ER) significantly affected (*P* < 0.10) the traits *per se* in the F_7_ lines. Of these eight SQs, five were associated with PH-related traits, which is consistent with the fact that broad-sense heritability was higher for PH-related traits than for the other traits. Additionally, we detected three SQs (associated with TL, TB, and LLA) that significantly affected (*P* < 0.10) the GCA for the traits in TCP2. *QSTL1-2* significantly affected (*P* < 0.10) the GCA for TL in TCP2, but not significantly affected (*P* < 0.10) TL in the F_7_ lines (Table 4, the fifth line). The additive effects of SQs in the MP were all consistently followed by the effects on traits *per se* in the F_7_ lines and the GCA effects in TCP2.

**Table 4:** **General combining ability of stable quantitative trait loci in the testcross population TCP2**.

**Trait**	**SQ**	**Locus Name**	**Effect in MP^a^**	**Effect in F_7_ lines^b^**	**Effect in TCP2^c^**
PH	QSPH1	PZE-101213558	N/M	N^*^	N^NS^
PH	QSPH2	SYN5428	N	N^*^	N^NS^
PH	QSPH5	PZE-105128589	N/M	N^*^	N^NS^
EH	QSEH5	PZE-105138426	N/M	N^**^	N^NS^
TL	QSTL1-2	PZE-101219724	N/M	N^NS^	N^+^
TL	QSTL2	SYN5428	N/M	N^**^	N^**^
TB	QSTB2	PZE-102049280	P/M	P^*^	P^NS^
LLA	QSLLA2	PZE-102178263	N/M	N^**^	N^*^
ER	QSER3	PZE-103022844	N/M	N^+^	N^NS^

*The abbreviations PH, EH, TL, TB, LLA, and ER refer to plant height, ear height, tassel length, tassel branch number, leaf length above the primary ear, and ear row number, respectively*.

a *N indicates that the additive effect of alleles contributed by R08 increased the trait value. P indicates that the additive effect of alleles contributed by Ye478 increased the trait value. M indicates a major QTL*.

b *N indicates that the alleles contributed by R08 increased the trait per se value. P indicates that the alleles contributed by Ye478 increased the trait per se value*.

c *N indicates that the alleles contributed by R08 increased the additive effect value for the trait. P indicates that the alleles contributed by Ye478 increased the additive effect value for the trait*.

+, *, and ** *indicate significance at P < 0.10, 0.05, or 0.01, respectively*.

*NS indicates that the effect was not significant*.

## Discussion

### Pyramiding of favorable alleles in different types of maize foundation parents

Different types of maize foundation parents are the result of breeding for diversity. Determining the genetic basis for multiple traits in different maize foundation parents may provide useful information for breeding programs. In this study, QTL mapping was used to analyze 15 traits in 266 F_2:3_ families derived from two maize foundation parents across six environments. We used single trait analysis rather than multiple trait analysis in this study, because, multiple traits analysis models might not unbiased due to the increase in the number of parameters to be estimated as a result of large number of traits and environments in fewer lines, and multiple traits analysis model can have lower power to identify QTLs that have effects on only a small subset of traits when compared to the single trait analysis model, due to greater genome-wide threshold in the multiple trait analysis model (Malosetti et al., 2008; Silva et al., 2012; Alimi et al., 2013). Our results indicated that Ye478 carries more favorable alleles associated with most plant type-related traits than R08. This result was most obvious for SQs and important QTLs. For PH, more than 95% of favorable alleles, 95% of favorable additive effects, and all favorable alleles for SQs and important QTLs were from Ye478. More than 50% of favorable alleles related to ED, ER, and KW were from Ye478, but most favorable alleles associated with EL were derived from R08. Several studies determined that there are more favorable alleles that can reduce PH or EH in Ye478 than in other inbred lines (including other elite lines) (Zhang et al., 2007; Yang X. J. et al., 2008; Zhang Y. et al., 2010; Zhao et al., 2011). This is consistent with the results for foundation parents developed from other germplasms. For instance, Steinhoff et al. (2012) reported that European foundation parents possess diverse alleles. Buckler et al. (2009) and Tian et al. (2011) concluded that B73 carries several favorable alleles and most of the QTLs contain more than two alleles.

**Figure 2:**
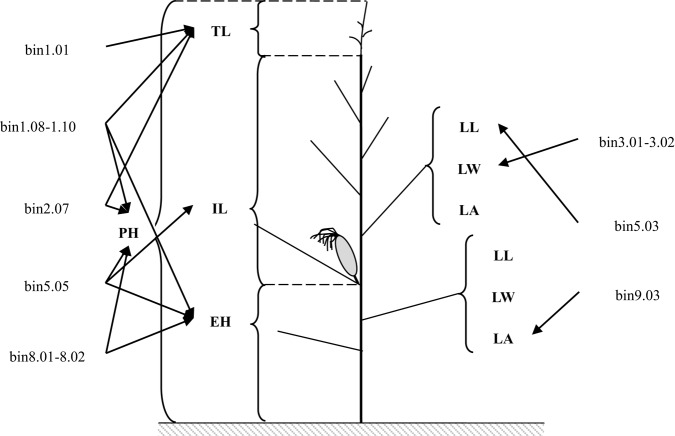
**Genomic regions containing genes affecting specific plant organs**. The abbreviations PH, EH, IL, TL, LL, LW, and LA refer to plant height, ear height, internode length above the primary ear, tassel length, leaf length, leaf width, and leaf angle above the primary ear, respectively.

The maize ideotype is important for breeding. Plants should consist of vertical canopies in which there are gradual increases in leaf angles and leaf areas from the top of the canopy to the bottom (Mock and Pearce, 1975; Stöckle and Kemanian, 2009). The five genomic regions associated with PH-related traits and the three genomic regions associated with leaf architecture-related traits identified in this study exhibited organ-specificity for similar traits (Figure 2). *QSPH1, QSEH1*, and *QSTL1-1*, which affected PH, EH, and TL, respectively, were detected in bin 1.08–1.10. This genomic region did not include the QTL for IL. *QSPH2* and *QSTL2*, which influenced PH and TL, respectively, were detected in bin 2.07. Additionally, *QSPH8* (associated with PH) and *QSEH8* (associated with EH) were detected in bin 8.01–8.02. These two genomic regions included organ-specific genes that affected only one PH-related trait. For leaf architecture-related traits, *QSLLA5* (for LLA) was detected in bin 5.03, *QSLWA3* (for LWA) was detected in bin 3.01–3.02, and *QSLAB9* (for LAB) was detected in bin 9.03. These three genomic regions included organ-specific genes that only influenced leaf components. Zhang J. et al. (2010), Liu Z. et al. (2014), and Ku et al. (2015) reported that the genetic basis for plant type in different organs were different. The decreased PH of Ye478 was mainly due to the pyramiding of the favorable alleles for EH, and the favorable alleles that decrease leaf areas above the primary ear or increase leaf angles below the primary ear. Therefore, Ye478 contained favorable ideotype alleles, which may explain why it is widely utilized in China. The pyramiding of favorable alleles has also been observed in other foundation parents. For example, Peiffer et al. (2014) revealed that B73 carries favorable alleles related to EH and PH. Additionally, Lima et al. (2006) determined that an allele located in bin 3.06 –3.07 from a Brazilian tropical germplasm (L-02-03D) decreased PH by reducing EH.

Maize is widely grown in China, including in Tibet. Based on the history of cultivation, temperature, photoperiod response, rainfall, frost-free period, and cropping system, the maize producing regions in China can be divided into the following six zones: The north zone, the Huanghuaihai zone, the southwest zone, the south zone, the northwest zone, and the Qinghai–Tibet altiplano (Li, 2009; Zhang and Bonjean, 2010). The summer maize varieties grown in the Huanghuaihai zone exhibits high yield potentials, lodging resistance, and tolerance to high plant densities, while the maize varieties grown in the southwest zone exhibits large ears (Chen F. B. et al., 2007; Wang and Li, 2010; Zhang and Bonjean, 2010; Ci et al., 2012). The significant differences in plant type-related traits between Ye478 and R08 resulted from the differences in the ideotype of the maize varieties grown in the Yellow and Huai River zone and the southwest zone. In the major maize-producing areas (i.e., United States, China, and other countries), there has been a trend toward the breeding of varieties adapted to high plant densities and resistant to lodging (Duvick, 2005; Tollenaar and Lee, 2011; Ci et al., 2012; Ma et al., 2014). Therefore, foundation parents should possess numerous favorable alleles for plant type-related traits to accommodate this trend. There has also been a focus on the development of inbred lines with increased grain yield (Duvick, 2005; Ci et al., 2011; Lauer et al., 2012). Consequently, increased grain yield in maize hybrids have been mostly attributed to the greater grain yield in the parents, and not because of heterosis (Duvick, 2005; Ci et al., 2011). Therefore, our findings and those from previous studies suggest that foundation parents should carry several favorable alleles related to yield-related traits. Additionally, different types of foundation parents may accumulate specific favorable alleles that are appropriate for particular environments (Peng et al., 2011; Li et al., 2013; Guo et al., 2014). This may explain the observed heterosis associated with grain yield among different types of foundation parents (Goff, 2011; Goff and Zhang, 2013).

### Improvement of Ye478

The inbred line Ye478 is highly adaptable and exhibits a high combining ability and excellent plant architecture. A number of inbred lines descended from Ye478 perform well in different environments, and thus are widely used in multiple maize-producing regions in China (Li and Wang, 2010). In the present study, TCP1 was used to study the relationship between Ye478 and its related lines. The performance of traits *per se* and the GCA for traits for the Ye478-related lines revealed that Ye478 inherited advantageous traits from U8112 and Shen5003. Additionally, the typical Ye478 characteristics had been transmitted to subsequent generations' derivatives, resulting in descendants with similar agronomic traits (Liu Y. H. et al., 2011). The values for PH, EH, and LAA *per se* and the GCA for these traits were lower in Zheng58 and Zao48 than in Ye478, thus variations from Zheng58 and Zao48 better adapted to high plant densities. With K22 as a parental line, more than 10 descended inbred lines and eight important hybrids have been released in Shaanxi province and Inner Mongolia of China. The results for traits *per se* and the GCA for traits for K22 differed from those of Ye478 because the breeding of K22 focused on yield per plant and adaptation to the arid environments of northwest China (Li et al., 2004; Supplemental Table 1).

Maize is an example of one of the most successful use of heterosis in crop production. The development and improvement of foundation parents has been closely associated with heterosis (Teng et al., 2004; Troyer, 2004). U8112 was an excellent inbred line from the improved Reid group, and there was a considerable genetic distance between U8112 and other representative lines of different heterotic groups (Shi, 2007; Wu et al., 2014). U8112, Ye478, and Zheng58 belong to the same main subgroup, and all have been frequently used in Chinese maize breeding programs (Supplemental Table 1). In the present study, the heterotic patterns of Ye478 were inherited from U8112, and were transmitted to Zheng58. This explains why Ye478 and Zheng58 can be used in the development of hybrids by using lines from different heterotic groups to as another parent. This conclusion is consistent with maize breeding practices and the genetic characterization of elite maize lines (Zhao, 2003; Nelson et al., 2008, 2016). The pattern of improvement and use of Ye478 can also be observed for B73, Mo17, and other foundation parents. Godshalk and Kauffmann (1994) reported that B73 and its descendants are high yielding lines with a high GCA for yield. Nelson et al. (2008) determined that the largest genetic distance among expired United States Plant Variety Protection maize inbred lines was between B73 and Mo17, and their descended inbred lines had similar genetic distances.

Analyses of SGRs revealed that Ye478 had inherited the semi-dwarfism characteristic from Shen5003, and inherited the compact appearance trait from U8112. We observed that SGR1, SGR2, and SGR3 were associated with PH, and were highly conserved in Ye478 descendants. Weng et al. (2011) detected a dwarf locus in bin 5.05–5.06, which was consistent with our results regarding *QSPH5*/SG3. The locus was derived from Shen5003, and was retained in Ye478 and Zheng58. Because SGR1, SGR3, and SGR4 also affect EH, there were no significant differences in EH between Ye478 and its descendants. There were half of SGRs were consistent between PH and IL (or TL), and this might be the reason of there were no significant differences in IL (or TL) between Ye478 and some of its descendants. SGR2 was closely linked to a major QTL for LLA. Therefore, this genomic region was highly conserved in Ye478 descendants and LLA in Ye478's descendants all change no more than 11% of the Ye478. In contrast, SGR4–SGR8 had been replaced in at least one Ye478 descendant. SGR5, SGR6 and SGR7 were closely linked to the QTLs for LWA, and thus the change trend of K22, Zao48, and Zheng58 were consistent in LAA and LWA. Because SGR6 was closely linked to a major QTL for EL, and this genomic region was replaced in Zheng58, the EL *per se* in Zheng58 changed more than 10% of the Ye478. Lai et al. (2010) determined that there were considerable differences among the genomes of the Ye478-related lines. Jiao et al. (2012) concluded that rare alleles were continuously accumulating in foundation parents during maize breeding. Therefore, the changes in performance and GCA in the Ye478 descendants were because these lines inherited favorable alleles from Ye478 and accumulated new favorable alleles from other parents.

### Potential utility of QTLs for traits *per se* to improve the GCA of foundation parents

The development of single cross hybrids is the most important use of maize inbred lines. Therefore, a high GCA is crucial for foundation parents (Rojas and Sprague, 1952; Li and Wang, 2010). However, estimating GCA is expensive and time-consuming because multiple hybrid combinations are required and repeated field trials are needed. Marker-assisted selection (MAS) represents a viable and cost-effective way to improve traits that are difficult to evaluate and/or are expensive to characterize (Yousef and Juvik, 2001; Eathington et al., 2007; Qi et al., 2013). The efficiency of MAS during plant breeding is affected by the number of QTLs, the effects of QTLs, and their stability in multiple environments (Bernardo, 2008).

In this study, we detected eight SQs that significantly affected traits *per se* in F_7_ families. Additionally, three SQs affecting the GCA for traits were identified in TCP2. *QSTL2* and *QSLLA* were two QTLs that significantly affected traits *per se* and the GCA for traits. Seven SQs influenced either traits *per se* or the GCA for traits. These results indicated the molecular regulation of traits *per se* differed from that of the GCA for traits. Several studies have been conducted on QTL mapping of traits *per se* and the GCA for traits. Lv et al. (2012) analyzed the consistency of QTLs between traits *per se* and the GCA for traits in inbred lines using a subset of maize introgression lines. They pointed out that the molecular basis for the GCA for traits and traits *per se* was different despite the fact that there were consistent QTLs. Qu et al. (2012) detected QTLs in the traits *per se* dataset and GCA datasets, and found that there was a lack of consistency. Additionally, they also observed that the QTL for GCA could be stably detected in different testcross populations. Huang et al. (2013) identified a few common QTLs for GCA and for traits *per se*. These QTLs were mainly related to row number and PH, and these two traits may be highly heritable. These results revealed the complexity of the molecular regulation of GCA.

Although the QTLs for traits *per se* could not be used to predict the GCA for traits, it is noteworthy that the additive effects of SQs in the MP were all consistently followed by the effects on traits *per se* in the F_7_ lines and the GCA for traits in TCP2. The tester used was a complex factor affecting the analysis of the GCA because the GCA is influenced by the genetic background of the tester (Austin et al., 2000). Several studies found that QTL effects were approximate across multi-tester populations, even though there was a lack of consistency among QTLs between tester populations (Melchinger et al., 1998; Austin et al., 2000; Frascaroli et al., 2009). Foundation parents with excellent agronomic characteristics, a high GCA for yield-related traits, and carried several favorable alleles (Jiao et al., 2012). Therefore, the favorable alleles detected for traits *per se* or GCA for traits may be useful in improving traits *per se* and GCA for traits in inbred lines. Multi-parent cross design populations, including the Nested Association Mapping (Buckler et al., 2009) and Multi-parent Advanced Generation Inter-Cross (Dell'Acqua et al., 2015) populations, with superior genetic diversity and high mapping power can be used to detect favorable alleles. The use of multiple independent populations, with a flexible and manageable design, is a cost-effective way to integrate widely available genetic resources to detect favorable alleles (Xiao et al., 2016). Previous QTL studies in various mapping populations have provided abundant information for identifying favorable alleles by meta-analysis. To more effectively develop new foundation parents and improve old foundation parents, the integration of all QTL information to enable the detection of favorable alleles for MAS is required. Breeders may use genome-wide selection, which is superior to MAS, to improve foundation parents (Bernardo and Yu, 2007). Additionally, breeders could use the *Mapping As You Go* approach to continually revise estimates of QTL allele effects by remapping new elite germplasm generated over cycles of selection to ensure that QTL estimates remain relevant to the current set of germplasm in the breeding program (Podlich et al., 2004).

## Author contributions

YH, YL, and XBH designed the study. XBH, QX, SB, WC, and XQH completed the experiments. YHL, XBH, and JZ analyzed the data. YHL and XBH prepared the manuscript, and all authors read, and approved the manuscript.

### Conflict of interest statement

The authors declare that the research was conducted in the absence of any commercial or financial relationships that could be construed as a potential conflict of interest.
